# A case report of carcinoma of uterine cervix throwing heterochronous metastasis to the skin, spleen, and pancreas: the role of multimodality treatment approach

**DOI:** 10.1186/s43046-019-0009-9

**Published:** 2019-12-16

**Authors:** P. K. Gupta, P. Lal, A. Tiwari

**Affiliations:** 1 Department of Radiation Oncology, Super Speciality Cancer Institute and Hospital, C.G. City, Lucknow, Uttar Pradesh 226002 India; 2 Department of Radiotherapy, Sanjay Gandhi Postgraduate Institute of Medical Sciences, Lucknow, Uttar Pradesh 226014 India; 3 Royal Cancer Institute and Research Centre, Kanpur, Uttar Pradesh India

**Keywords:** Carcinoma of the uterine cervix, Cutaneous, Splenic, Pancreatic metastasis

## Abstract

**Background:**

Cancer of cervix often fails locally and/or within the pelvis. One to two percent of cervical squamous cell carcinoma patients have lung metastases at presentation, and 5–35% develop pulmonary metastases later on. Common sites of metastases are the liver, bone, and bowel. We report a rare case presentation of cervical squamous cell cancer where heterochronous metastasis occurred in the skin, spleen, and pancreas without loco-regional recurrence and skipping of visceral organs such as the lung, liver, and brain.

**Case presentation:**

A 55-year-old, postmenopausal lady presented with a complaint of bleeding of the vagina for 2 months duration. Cervical biopsy revealed squamous cell carcinoma of the cervix, and she was staged as a case of FIGO stage IIIB. She received external beam-beam radiotherapy of 50 Gy in 25 fractions along with concurrent weekly cisplatin at 35 mg/m^2^ followed by 3 fractions of intracavitary brachytherapy of 6 Gy each. After 30 months of follow-up, she presented with a skin lesion of 6 × 5 cm^2^ in the infrascapular region for 2 months duration. Biopsy revealed metastatic squamous cell carcinoma. Her metastatic work-up revealed no other lesions. Palliative radiotherapy to local site of 8 Gy in single fraction was delivered. The lesion disappeared within 4 weeks. She was given 6 cycles of cisplatin and paclitaxel salvage chemotherapy. After 30 months of follow-up, she presented with a skin lesion of 6 × 5 cm^2^ in the infrascapular region. Biopsy revealed metastatic squamous cell carcinoma. Her metastatic work-up revealed no other lesions. Palliative radiotherapy to the local site was planned, and a dose of 8 Gy in a single fraction was delivered. The lesion disappeared within 4 weeks. She was given 6 cycles of cisplatin and paclitaxel salvage chemotherapy. Six months after the completion of salvage therapy, she reported with the complaints of recurrent bouts of hematemesis and melena. Her CECT scan revealed 2 × 1.5 cm^2^ growth in the body of the pancreas and a subcentric splenic hilum node. She underwent open splenectomy with distal pancreatectomy. Histopathology report showed metastatic infiltration in pancreatic tissue by squamous cell carcinoma and one metastatic node in the splenic hilum. Post-treatment, 6 months, the patient was asymptomatic with no recurrence.

**Conclusions:**

This is a rare heterochronous metastatic presentation of cervical cancer without loco-regional recurrence and visceral organs such as the lung, liver, and brain. The optimal treatment remains undefined for these patients. Multimodality treatment is necessary to manage the patients.

## Background

In cervical cancer, cutaneous metastasis without local recurrence is rare. Cancer of cervix often fails locally and/or within the pelvis. One to two percent of cervical squamous cell carcinoma patients have lung metastases at presentation, and 5–35% develop pulmonary metastases later on [1–3]. Common sites of metastases are the liver, bone, and bowel [1–3]. Skin involvement as an isolated site of spread from the cervix has been reported in 0.1–2.0% cases [1–4]. Furthermore, epithelial cancers metastasizing to the spleen are very rare, and available literature is scarce. Until now, 22 cases of cervical cancer with splenic metastases have been documented in the literature with a range of 3–6 cases of isolated splenic metastases. Patients with splenic metastasis have a poor prognosis. Surgery is indicated in symptomatic patients like patients having a splenic rupture. Uterine cervical cancer with pancreatic metastasis is extremely rare with only few cases reported. We report a rare presentation of cervical cancer which had a rather unusual spread pattern, i.e., it first spread to the skin and then subsequently to other visceral organs, while it skipped the regional lymphatics and common visceral organs such as the lung, liver, and brain.

## Case presentation

A 55-year-old, postmenopausal lady presented to the department of radiotherapy with a complaint of bleeding of the vagina for 2 months duration. On examination, she was obese with Karnofsky Performance Status of 90. Per abdomen, examination revealed hepatomegaly just below the costal margin. Per speculum and per vaginal examination showed an infiltrative growth at the site of the cervix involving anterior and posterior fornices. Bilateral parametrium was involved up to the lateral pelvic wall. Cervical biopsy revealed squamous cell carcinoma of the cervix. On investigations, her biochemical, hematological, and radiological examinations including ultrasound of abdomen and pelvis and cystoscopic evaluation were normal. She was staged as a case of FIGO stage IIIB carcinoma of the uterine cervix, as per the FIGO 2009 staging system.

The patient was planned for chemoradiation. She received whole-pelvic external beam radiotherapy of 50 Gy in 25 fractions by four-field box technique along with concurrent weekly cisplatin at 35 mg/m^2^ capped at 50 mg for 5 weeks. This was followed by 3 fractions of intracavitary brachytherapy of 6 Gy each, delivered 1 week apart. She tolerated the treatment well and had a complete clinical and radiological response and was kept on regular follow-up thereafter.

After 30 months of follow-up, she presented with a skin lesion of 6 × 5 cm^2^ in the infrascapular region for 2 months duration. Biopsy taken from this site revealed metastatic squamous cell carcinoma. Her metastatic work-up (including a CECT scan of the abdomen and pelvis) revealed no other lesions. Palliative radiotherapy to the local site was planned, and a dose of 8 Gy in a single fraction was delivered. The lesion disappeared within 4 weeks. She was given 6 cycles of cisplatin and paclitaxel salvage chemotherapy.

**Fig. 1:**
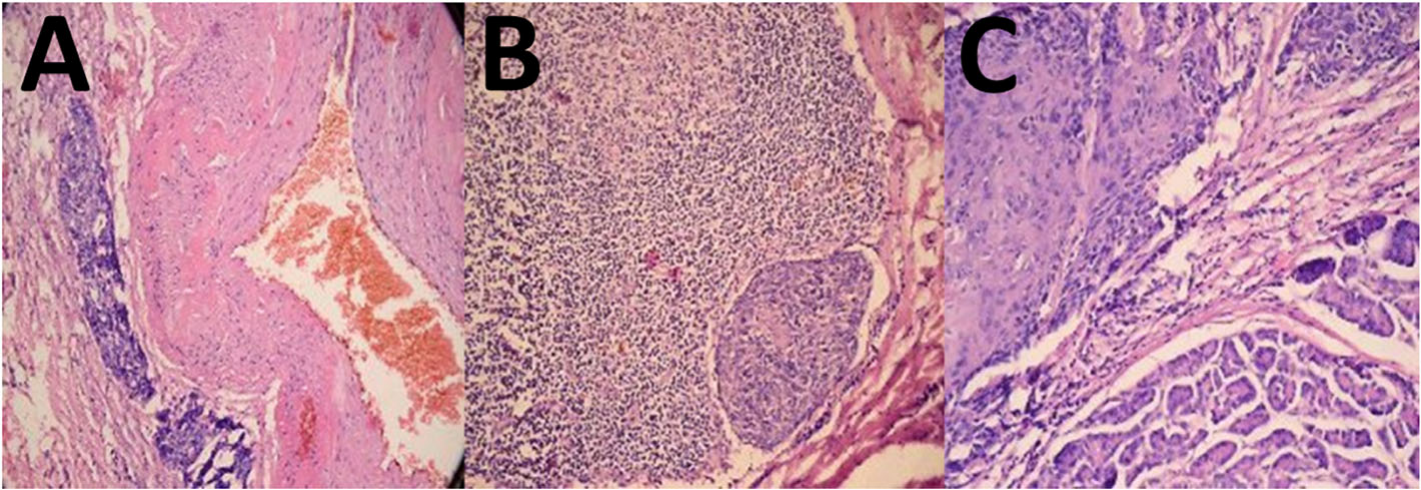
**a** Squamous cell carcinoma infiltrating the pancreas (H&E, × 20). **b** Splenic hilar lymph node with subcapsular tumor deposit (H&E, × 20). **c** Tumor in the vicinity of the splenic artery (H&E, × 20)

Six months after the completion of salvage therapy, she reported with the complaints of recurrent bouts of hematemesis and melena. Her local examination and metastatic work-up (including a CECT scan of the thorax, abdomen, and pelvis) showed no abnormal lesion. She was given 3 units of blood transfusion support for anemia and was referred to the department of gastromedicine for further evaluation. Upper gastro-endoscopic evaluation revealed isolated gastric varices. She underwent four sessions of injection glue (total dose, 11 ml). The patient however, continued to have recurrent small episodes of hematemesis and melena. She was re-evaluated by the medical and surgical gastroenterology teams and was considered for variceal surgery. Her repeat CECT scan revealed 2 × 1.5 cm^2^ growth in the body of the pancreas and a subcentric splenic hilum node. She underwent open splenectomy with distal pancreatectomy. Histopathology report showed metastatic infiltration in pancreatic tissue by squamous cell carcinoma and one node in the splenic hilum (Fig. 1). Post-treatment, 6 months follow-up, the patient was asymptomatic with no recurrence.

## Discussion

Skin involvement as an isolated site of spread from the cervix has been reported in 0.1–2.0% cases [4]. In a series of 695 patients, Brady et al. reported 1% incidence of skin metastasis while another larger series of 1190 patients reported 1.3% incidence of skin metastasis [4–6]. The site of skin metastasis frequency in decreasing order is the abdominal wall, vulva, and anterior chest wall [2]. Patients with adenocarcinoma and neuroendocrine undifferentiated tumors have a higher incidence of skin metastasis as compared to patients with squamous cell carcinoma [7]. Extirpation of the skin lesion followed by radiotherapy is the main treatment for these patients [5, 6, 8, 9].

Furthermore, epithelial cancers metastasizing to the spleen are very rare, and available literature is scarce. Splenic metastases from cervical carcinoma can be a part of a disseminated disease or can present as solitary metastases [10]. The splenic metastases incidence varies from 1.6 to 30%. Breast cancer, lung cancer, and melanoma are the most common primary malignancies to have splenic metastasis [11, 12]. Out of 2200 patients with cervical cancer, Carson et al. have reported distant metastases of 15.3% [2]. Brufman et al. reported the first case in 1977 [12]. Until now, 22 cases of cervical cancer with splenic metastases have been documented in the literature with a range of 3–6 cases of isolated splenic metastases [13–19]. Patients with splenic metastasis have a poor prognosis. Surgical excision is done in the symptomatic patients like patients having a splenic rupture.

The lung, breast, skin (melanoma), stomach, colorectum, kidney, and ovary are the most common primary malignancies presenting with pancreatic metastases [20–22]. Heterochronic metastasis is common. Uterine cervical cancer spreading to the pancreas is very rare with only few case reports available in the literature [23–26].

In patients presenting with a pancreatic mass with a previous history of malignancy, the possibility of a metastatic pancreatic tumor should be considered. In the present report, we describe a heterochronic metastasis from primary squamous cell cervical cancer, metastasizing to the pancreas subsequent to cutaneous and splenic metastasis.

## Conclusion

Heterochronous metastasis to the skin, spleen, and pancreas in squamous cell carcinoma of the uterine cervix is very rare. Oncologists should be vigilant during follow-up for cutaneous lesions; hematemesis can be the first sign of recurrence, and biopsy should be performed from any suspicious cutaneous or pancreatic lesion.

## Data Availability

Can be shared from the patient file, if required

## Abbreviations

CECT: Contrast-enhanced computed tomography
FIGO: International Federation of Gynecology and Obstetrics
